# Controversies Regarding the Psychometric Properties of the Brief COPE: The Case of the Brazilian-Portuguese Version “COPE Breve”

**DOI:** 10.1371/journal.pone.0152233

**Published:** 2016-03-23

**Authors:** Sarah V. Brasileiro, Mara R. C. A. Orsini, Julianna A. Cavalcante, Daniel Bartholomeu, José M. Montiel, Paulo S. S. Costa, Luciane R. Costa

**Affiliations:** 1 Programa de Pós-Graduação em Ciências da Saúde, Universidade Federal de Goiás (UFG), Goiânia, Goiás, Brazil; 2 Departamento de Psicologia, Faculdade de Educação, UFG, Goiânia, Goiás, Brazil; 3 Programa de Pós-Graduação em Odontologia, UFG, Goiânia, Goiás, Brazil; 4 Programa de Pós-Graduação em Psicologia Educacional, Centro Universitário Fundação Instituto de Ensino para Osasco (UniFIEO), Osasco, São Paulo, Brazil; 5 Programa de Pós-Graduação em Psicologia Educacional, UniFIEO, Osasco, São Paulo, Brazil; 6 Departamento de Pediatria, Faculdade de Medicina, UFG, Goiânia, Goiás, Brazil; 7 Departamento de Saúde Oral, Faculdade de Odontologia, UFG, Goiânia, Goiás, Brazil; Philipps University Marburg, GERMANY

## Abstract

The Brief Coping Orientation to Problems Experienced (COPE) inventory investigates the different ways in which people respond to stressful situations. Knowledge is lacking regarding the coping strategies and styles of people in developing countries, including Brazil. This study aimed to adapt and validate the Brief COPE to Brazilian Portuguese (named *COPE Breve*) by focusing on dispositional coping. For the cross-cultural adaptation, the original Brief COPE in English (28 items grouped into 14 subscales) was adapted according to a universalistic approach, following these steps: translation, synthesis, back-translation, analysis by an expert panel, and pretest with 30 participants. Then, 237 adults from the community health service responded to the *COPE Breve*. Psychometric analyses included reliability and exploratory factor analysis. Most of the 14 subscales from the original Brief COPE exhibited problems related to internal consistency. A Velicer's minimum average partial test (MAP) was performed and pointed out 3 factors. Exploratory factor analysis produced a revised 20-item version with a 3-factor solution: religion and positive reframing, distraction and external support. The psychometric properties of the *COPE Breve* with three factors were appropriate. Limitations of this study as well as suggestions for future studies are presented. The *COPE Breve* should be used in Brazilian clinics and investigations, but divergences in its psychometrics should be further explored in other contexts.

## Introduction

Disease is a stressful situation for both sick individuals and their caretakers. Within this context, coping comprises a set of continuously changing cognitive and behavioral strategies that individuals employ to deal with circumstances considered stressful for themselves and their environment [1].

The model of coping formulated by Folkman and Lazarus [2] is the most comprehensive and is based on four major assumptions: (a) coping is a process or interaction involving individuals and their environment; (b) the function of coping is to manage, rather than control or dominate stressful situations; (c) coping presupposes the notion of evaluation (i.e., how the phenomenon is perceived and cognitively interpreted in an individual’s mind); and (d) coping involves the mobilization of behavioral and cognitive efforts to manage (i.e., reduce, tolerate, and minimize) internal or external demands that arise from the interaction between individuals and their environment.

The actions, behaviors or thoughts developed to manage stressors are called coping strategies. These strategies are subsequently divided into emotion-focused and problem-focused types. Emotion-focused strategies seek to change the emotional status of the individual in order to reduce the unpleasant physical feelings associated with stress. In turn, problem-focused strategies tackle difficulties present within the individual’s interaction with his environment and can in turn guide the individual’s internal and external actions [3]. In this regard, coping “strategies” should be distinguished from coping “styles”. Strategies concern the cognitive or behavioral actions that are performed during a particular episode of stress; they are associated with situational factors and can change at any moment over the course of a stressful situation. Conversely, styles are related to an individual’s personality traits and dispositional factors. In other words, each individual develops typical patterns for handling stress (i.e., coping styles) that can influence his or her reactions in new situations [4].

Healthcare professionals should identify the coping styles of individuals to improve their ability to cope effectively with the sources of stress that they must face over the course of a disease [5]. Several instruments have been formulated for this purpose, and the Coping Orientation to Problems Experienced inventory (COPE), elaborated by Carver et al. [6] is frequently mentioned in the literature. The COPE Inventory consists of 15 subscales containing four items each. However, because the COPE is a 60-item questionnaire the magnitude of which is burdensome for respondents, Carver created the Brief COPE with only 28 items to solve the problems detected in the original version [7].

Validated versions of the Brief COPE in Spanish (Spain) [8], Korean (South Korea) [9], French (France) [10], Portuguese (Portugal) [11], Greek (Greece) [12], and Tamil (India) [13] are currently available. To the best of our knowledge, only two studies have reported the use of the Brief COPE with Brazilian people, and both applied a Portuguese (Portugal) adapted version of the Brief COPE Inventory for university students in Brazil [14,15]. In the first study [14], the authors reported that the Portuguese (Portugal) version had a 14-factor structure and valid factorial, convergent and discriminant characteristics in Portugal and Brazil, but a direct comparison of the Brief COPE scores between the two countries was not possible due to their variability; those authors consequently hypothesized that cultural and socioeconomic factors could have an influence on coping. Conversely, the second study [15] was not able to confirm the 14-factor structure initially proposed. As the Portuguese language and cultural aspects differ in many respects in Portugal and Brazil, it is possible that a Portuguese version for Brazilian Portuguese directly adapted from the original instrument could yield better results.

Some data regarding the instrument’s psychometric properties remain controversial. Carver in 1997 [7] applied the Brief COPE to 168 adults recovering from Hurricane Andrew and tested the situational coping format using a 4-point Likert scale. He found that the Brief COPE had a 9-factor structure (exploratory factor analysis with oblimin rotation) and Cronbach’s alpha for each of the 14 subscales from the full COPE ranged from 0.54 to 0.90 [7]. Fifteen years later, a systematic review focusing on the Brief COPE religion subscale, identified 38 out of 212 empirical studies using the Brief COPE that conducted an exploratory factor analysis on their own dataset. The author concluded that the diversity in the factorial structure found was frequently due to an inappropriate analytic technique [16]. As that study included studies published until 2009 [16], we report a few details on the persistently divergent factorial structure of the Brief COPE as exhibited in the studies published from 2010 to 2015 (Table 1).

**Table 1:** Summary of studies on psychometric properties of the Brief COPE published from 2010 to 2015.

Study	Participants	Coping format	Responses in Likert scale	Psychometric properties
Kapsou et al. (2010) [12]	1,127 Greek adults in different contexts	Dispositional	4-point	EFA: 8 factors; CFA: second 8-factor model with acceptable fit
Astorga et al. (2010) [17]	260 adults in Spain	Unclear	4-point	alpha (14 original subscales): 0.50 to 0.90; PCA: 11 factors; Second order PCA: 4 factors
Amoyal et al. (2011) [18]	362 hospitalized patients with major burn injuries, United States	Situational	4-point	EFA: 7 factors; alpha: 0.56 to 0.86
Kimemia et al. (2011) [19]	134 Kenyan caregivers for a family member living with HIV/AIDS	Situational	4-point	alpha (14 original subscales): 0.22 to 0.62; PCA: 5 factors
Snell et al. (2011) [20]	147 adults with mild traumatic brain injury, New Zealand	Dispositional	4-point	alpha (14 original subscales): 0.43 to 0.97; CFA (original 9 factors): less than satisfactory; EFA: 3 factors; alpha (3 factors): 0.77 to 0.84
Hur et al. (2012) [21]	4,736 female twins, United Kingdom	Dispositional; 2 new items on eating and exercising added to Brief COPE	4-point	CFA: 3 factors; alpha: 0.71 to 0.78
Krägeloh et al. (2012) [22]	616 undergraduate students, New Zealand	Dispositional	4-point	EFA: 4 factors; CFA: 3 factors
Montel et al. (2012) [23]	49 patients with amyotrophic lateral sclerosis, France	Situational	4-point	EFA: 3 factors; alpha: 0.71 to 0.84
Hooper et al. (2013) [24]	168 African American smokers	Situational, coping during attempts to quit over the past two weeks	4-point	PCA: 2 factors; alpha: 0.85 and 0.78
Mejorada et al. (2013) [25]	203 Mexican women with breast cancer undergoing radiotherapy	Situational, last 7 days	4-point	EFA: 7 factors with 17 items; alpha (7 factors): 0.62 to 0.91
Morán et al. (2014) [15]	899 Brazilian college students	Dispositional; Portuguese-Portugal version	4-point	EFA: 8 factors, item 5 eliminated; CFA: the 8 factors showed a good structure
Doron et al. (2014) [26]	2,187 (study 1) and 584 (study 2) French college students	Dispositional	4-point	CFA: 14 original subscales (problem with the self-distraction scale first item); alpha (14 subscales): four subscales < 0.60; hierarchical measurements: 5 higher-order dimensions
Maroco et al. (2014) [14]	1,573 college students from Portugal and Japan	Dispositional	5-point	CFA: 14 original subscales fitted; alpha (14 factors): 0.66 to 0.90
Mohanraj et al. (2015) [13]	299 Indian people living with HIV/AIDS	Situational (HIV)	4-point	CFA for the original 14 subscales: poor fit; EFA: 5 factors, 16-item scale; CFA for the 5 factors: very good model fit; alpha (5 factors): 0.60 to 0.91
Monzani et al. (2015) [27]	606 adults, Northern Italy	Situational: participants were asked to report how they coped with difficulty in achieving a goal in the last month	4-point	CFA: tested five models with different number of factors; the theoretically-based 14 subscales was the only one with good fit
Pozzi et al. (2015) [28]	148 adults with anxiety disorders	Dispositional	4-point	PCA: 9 factors; alpha (9 factors): 0.50 to 0.85
Ruiz et al. (2015) [29]	220 Hispanic and 186 Black pregnant women	Unclear; 4 questions from the Instrumental Support and Self-Blame were omitted	4-point	EFA: 2 factors; 1 venting item and 1 behavioral disengagement item were problematic; CFA: 2 factors; alpha (9 specific factors): 0.64 to 0.78
Su et al. (2015) [30]	258 Chinese adults living with HIV	Situational; coping in the last 3 months	4-point	Alpha (14 original factors): 0.50 to 0.90; CFA: previous suggested models with 2 or 3 factors did not fit; EFA: 6 factors

EFA, exploratory factor analysis; CFA, confirmatory factor analysis; alpha, Cronbach’s alpha; PCA, principal component analysis

Many Brazilians who have to cope with stressful health issues come from the underserved population. As the Brief COPE was not investigated in this context, and in view of the concerns mentioned above, the aims of the present study were twofold: 1) to translate and adapt the original Brief COPE to Brazilian Portuguese, and 2) to assess the psychometric properties of the adapted version including its factorial structure, focusing on the coping style (dispositional coping), in a sample of low-income adults.

## Materials and Methods

The Research Ethics Board of the Universidade Federal de Goiás, Brazil approved this study (protocol #363/2010). All participants were informed of the purpose and methods of the study and signed an informed consent form. All procedures were in accordance with the ethical standards of the Brazilian Ministry of Health, and with the 1964 Helsinki declaration and its later amendments.

### The Brief COPE

The original version of the Brief COPE [7] consists of the following 14 two-item scales: self-distraction, active coping, denial, substance use, use of emotional support, use of instrumental support, behavioral disengagement, venting, positive reframing, planning, humor, acceptance, religion, and self-blame. The questions are prefaced by a short introduction explaining that the respondents should indicate how they cope with problems in life (e.g., being diagnosed with a disease, school examinations, surgical operations, and job loss). The answers are scored from 1 (“I haven’t been doing this at all”) to 4 (“I’ve been doing this a lot”). The final result is expressed as a profile; the scales are not summed, and no total score is calculated. A principal component analysis with oblique rotation discovered nine factors with eigenvalues greater than 1. Cronbach’s alpha was higher than 0.6 for 11 of the 14 scales. The internal consistency values of the scales acceptance, denial, and venting were not adequate [7].

The Brief COPE can be used to evaluate both coping strategies and coping styles (dispositional coping). For the purpose of this study, dispositional coping was assessed.

### Participants

Volunteers were recruited from community health centers which are responsible for providing primary medical and dental care for low-income people from two cities in Midwest Brazil. For the pretest phase, 30 participants were selected using convenience sampling of a group of parents accompanying their children to a community dental service. They were asked to answer the first version of the adapted Brief COPE and comment on the inventory.

Regarding the psychometric tests, 237 adults who accompanied children to dental care completed the *COPE Breve*; 175 were the mothers (73.8%), 28 were the fathers (11.8%), and 34 (14.4%) had other types of relationships with the children. One of two trained examiners individually administered the *COPE Breve* to each participant. The participants were informed about the aim of the evaluation and were instructed on how to respond to the *COPE Breve*; specifically, they were first asked to identify a stressful situation, such as being diagnosed with a disease, undergoing surgery, job loss, or the death of someone close (dispositional coping). Then, they were instructed to score each item by writing the corresponding number (as explained above) between the brackets.

The sampling was non-probability because the aim was not to obtain a representative sample but to improve the evaluation of the instrument’s psychometric properties. The sample size was estimated based on the number of individuals needed to perform a factor analysis, and including the responses of at least five participants to each item in the questionnaire [31].

### Translation and cross-cultural adaptation of the Brief COPE

Initially, Charles S. Carver (the author of the Brief COPE) was informed of the aims of the present stud and authorized the creation of the Brazilian-Portuguese version of the questionnaire. The following five steps, similar to those suggested by Beaton et al. [32], were used for the cross-cultural adaptation: translation, synthesis, back-translation, analysis by an expert panel, and pretest.

Two native Brazilian-Portuguese speakers independently translated the items of the Brief COPE English version into Portuguese. To ensure conceptual equivalence from a clinical perspective, one of the translators was acquainted with the concepts addressed in the Brief COPE. The other translator was not informed of the aims of the study so that a purely literal translation could be produced. Both translators and one examiner synthesized the results of the translation.

Next, two native English speakers with no healthcare training and who were not informed of the aims of the study independently back-translated into English the Portuguese version which resulted from the synthesis. The original and back-translated versions were then compared to establish whether the latter represented the same content as the former. Discrepancies were documented and analyzed by an expert panel that included three healthcare professionals (a psychologist, a pediatrician, and a pediatric dentist), one professional with a degree in literature, and two translators. The panel established the equivalence between the original and target versions relative to the following four criteria: semantic equivalence, idiomatic equivalence, experiential equivalence, and conceptual equivalence.

Thirty volunteers, who were instructed to make suggestions to improve their understanding of the questions, completed the resulting version. The aim of this step was to establish whether the respondents understood the meaning of the questions and whether they answered them correctly, to ensure that the adapted version retained its equivalence in an applied situation. Following the pretest, the instrument was qualitatively evaluated to assess whether the respondents had any questions related to it, and whether the expert panel needed to meet again to change the questions.

The resulting instrument adapted for Brazilian Portuguese was named the *COPE Breve* (S1 Text).

### Exploratory factor analysis

We performed an exploratory factor analysis (EFA), a principal axis factoring extraction method by using SPSS v. 21. Because all of the coping strategies included in the *COPE Breve* might have been correlated with the others, we chose the direct oblimin (oblique) rotation as it is more suitable for life sciences [33]. The solutions were assessed using a scree plot, the Kaiser criterion, a Velicer's minimum average partial test (MAP) and parallel analysis (using R software). The Kaiser-Meyer-Olkin (KMO) index was calculated to measure sample size adequacy, and the values were categorized as follows: fair, 0.5–0.7; good, 0.7–0.8; very good, 0.8–0.9; and excellent, > 0.9 [34].

### Reliability

Twenty percent of the total sample (n = 24) completed the *COPE Breve* twice over a 3 to 4-week interval to assess test-retest reliability (tested by paired samples t-test). Effect sizes were calculated by Cohen´s d. The intraclass correlation coefficients (ICC) for the scale scores reported were calculated. The degree of reliability was classified based on ICC values as follows: weak, ≤ 0.40; moderate, 0.41–0.60; good, 0.61–0.80; and excellent, 0.81–1.00 [18]. The internal consistency for the scales was assessed using Cronbach’s alpha; a value of 0.60 was selected as the cutoff point for acceptability [35]. These analyses were performed using IBM SPSS v. 21.

## Results

### Cross-cultural adaptation

Because the Brief COPE items are clear, the language adaptation did not pose significant difficulties. The word “operation” in the questionnaire’s introduction was changed to “situation” since it encompasses several circumstances including operations, surgery, treatments, and other difficulties. The personal pronoun “I” (*eu*) was removed from the beginning of statements to standardize the questionnaire format. No problems were found with regard to the back-translation; however, changes were made to later steps.

A few changes in wording were needed to ensure semantic equivalence. Some words were replaced in the introduction: *iria* (you were going to have) with *teria que* (you would have to); *questionam* (ask) with *perguntam* (ask) and *em especial* (particular) with *específica* (specific). Answer options 1 and 3 (“I haven’t been doing this at all” and “I’ve been doing this a medium amount”) were rewritten as *Não tenho feito de jeito nenhum* (“No way I’ve been doing that”) and *Tenho feito mais ou menos* (“I’ve been doing it more or less”). With regard to grammar, item 13 “I’ve been criticizing myself” was translated as *Tenho me autocriticado(a)* (“I’ve been self-criticizing myself”); however, the expert panel decided to remove the prefix “self” so that the final text stated, *Tenho me criticado(a)* (“I’ve been criticizing myself”).

No changes were required for any item following the application of the preliminary version (pretest). Only some alterations to the questionnaire format were made based upon the suggestions to add brackets at the end of each statement for writing the assigned score, and to shorten the introduction for the sake of clarity and succinctness (S1 Text).

### Exploratory factor analysis

First, we verified the postulated 14-factor structure with a CFA, which presented poor fit indexes; therefore, we opted for the EFA model. The sample size met the minimum requirement to perform factor analysis [31, 36], as well to produce a good KMO value (0.702) and a satisfactory Bartlett’s sphericity test (X^2^ = 1,159.72, DF = 190, P < 0.001), indicating that the *COPE Breve* items had good factorability with moderate results.

**Fig 1:**
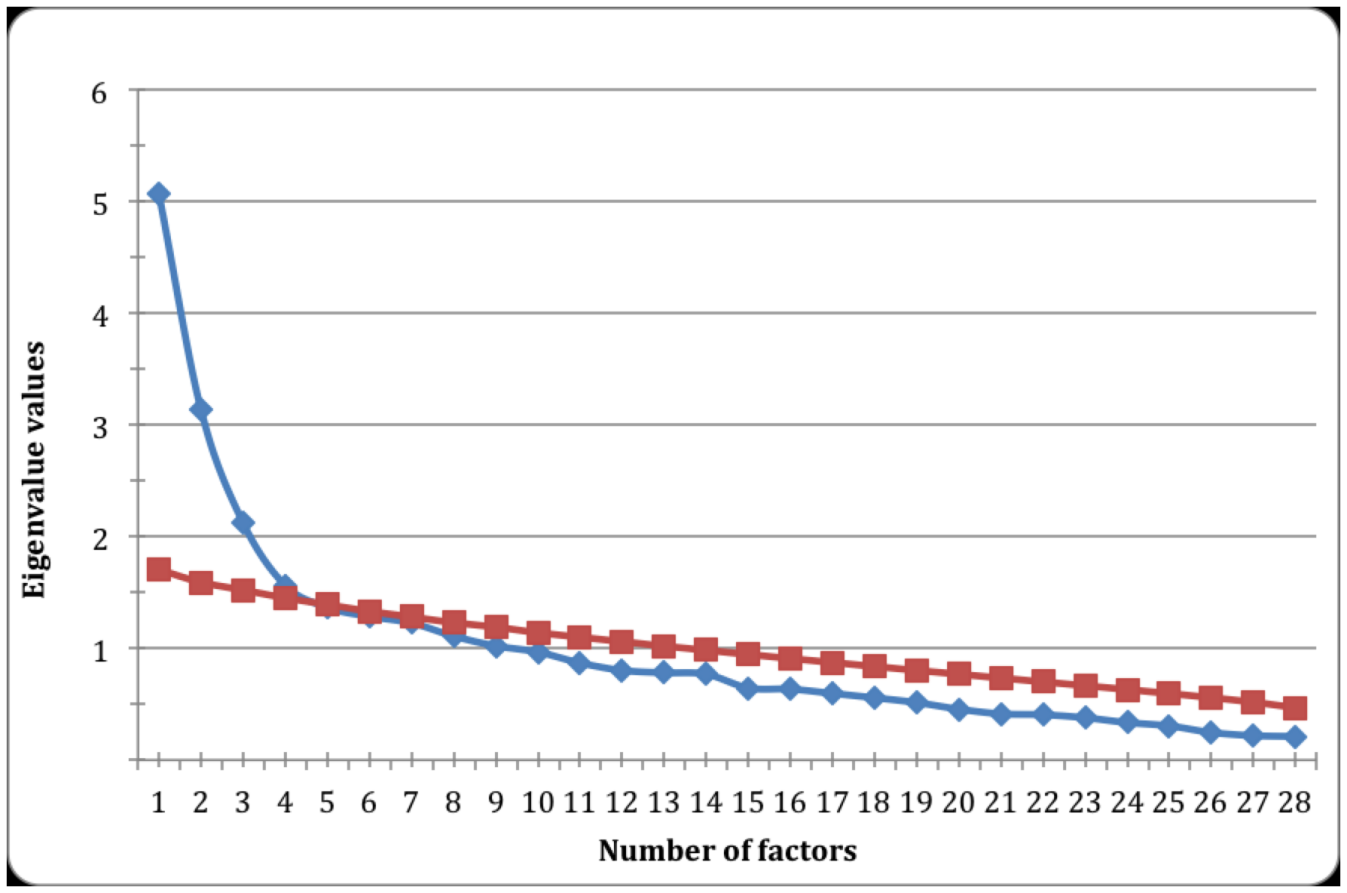
A scree plot representing the observed and random eigenvalues obtained in a parallel analysis of *COPE Breve*. Blue: observed eigenvalues; red: random eigenvalues.

The scree plot (Fig 1) showed the greatest discontinuity in the curve corresponding to the third factor, suggesting a possible 3-factor structure of the instrument [37]. According to the Kaiser criterion, only factors with eigenvalues larger than one should be retained, thus indicating that around eight factors should be retained here. For the parallel analysis, actual eigenvalues less than, or equal to, the parallel average random eigenvalues are attributable to sampling errors [38], so up to four factors ought to be retained. Also, a MAP test was performed and pointed out 3 factors, with a smallest average squared correlation of 0.015. Therefore, we analyzed a 3-factor structure for the Brazilian version of the Brief COPE.

The 3-factor structure best represented the theoretical constructs; the pattern and structure matrices are presented in Table 2. Importantly, the complex items were included in the factors with greater factor loadings.

**Table 2:** *COPE Breve* factors obtained using a principal axis factoring method with direct oblimin rotation (pattern and structure matrix).*

*COPE Breve* items (number as in the original Brief COPE)I’ve been…	Pattern Matrix			Structure Matrix			
	1	2	3	1	2	3	h^2^
(22) … trying to find comfort in my religion or spiritual beliefs	.643			.650			0.427
(24) … learning to live with it	.578			.604	.318		0.427
(7) … taking action to try to make the situation better	.554			.541			0.328
(27) … praying or meditating	.532			.530			0.320
(12) … trying to see it in a different light to make it seem more positive	.499			.515			0.327
(20) … accepting the reality of what has happened	.496			.497			0.266
(25) … thinking hard about what steps to take	.488			.495	.334		0.332
(17) … looking for something good in what is happening	.470			.487			0.247
(14) … trying to come up with a strategy about what to do	.399			.404			0.169
(11) … using alcohol or other drugs to help me get through it		.591			.577		0.279
(19) … doing something to think about it less, such as going to movies, watching TV, reading, daydreaming, sleeping, or shopping		.568			.576		0.343
(4) … using alcohol or other drugs to make myself feel better		.507			.502		0.373
(18) … making jokes about it		.456			.475		0.253
(1) … turning to work or other activities to take my mind off things		.452			.468		0.259
(21) … expressing my negative feelings		.422			.430		0.192
(6) … giving up trying to cope		.360			.362		0.132
(10) … receiving help and advice from other people	.337		.747	.413		.781	0.722
(26) … blaming myself for things that have happened			.515			.526	0.284
(5) … receiving emotional support from others			.463			.493	0.306
(28) … making fun of the situation			.397			.393	0.195
Eigenvalues	3.91		2.43	1.77			

* Eight items did not reach a minimum factor load of 0.3 and were excluded: (2) I've been concentrating my efforts on doing something about the situation, (3) I've been saying to myself, "This isn't real”, (8) I've been refusing to believe that it has happened, (9) I've been saying things to let my unpleasant feelings escape, (13) I’ve been criticizing myself, (15) I’ve been receiving comfort and understanding from someone, (16) I’ve been giving up trying to deal with it, (23) I’ve been trying to obtain advice or help from other people about what to do.

The factor correlations ranged from 0.104 to 0.127. The structural matrix presents the correlations between variables and factors and considers the unique variance between variables and factors and the correlation between them (the pattern matrix shows only the unique contribution of each variable to each factor). If the correlation between factors is high, it is difficult to distinguish which variables are only associated with each factor matrix [36]. Also, Thompson [39] recommends the inclusion of this matrix, emphasizing that the exclusion of these coefficients results in the loss of significant correlations of variables with factors. After analyzing these coefficients (Table 2), we have ascertained that most items had factor loadings in more than one dimension, but were more strongly associated with a particular one of them.

The communalities found by all items except one had indexes below 0.50, the recommended threshold [36]. MacCallum et al. [40] emphasize that when communalities are low, the role of sample size and six or seven indicators per factor and a low number of factors are important aspects in the recovering of the population factors. When the communalities are consistently below this value (as was the case in this study), in order to ensure a good recovery of population factors, the overdetermination of factors and large samples (over 100 subjects) are required. In this study, the structure identified three factors with a sample size over 100 subjects and only one factor with less than seven items, thereby fulfilling the criteria of MacCallum et al. [40], and suggesting the possibility of a good estimation of population factors.

The first of these factors was named “religion and positive reframing” because it comprised items from both scales, in addition to items both from acceptance and from planning, and one item from active coping [7]; it explained 19.59% of the variance. This factor included nine items that corresponded to strategies involving cognitive and behavioral control of situations and the use of religion to cope with stress.

The second factor explained 12.18% of the variance and included seven items. It was named “distraction” because it consisted of items both from the substance use and self-distraction subscales, as well as one item from each of the subscales of humor, venting and behavioral disengagement [7]. The use of these strategies suggests that individuals try to shift the focus of their attention away from stressful events to the point that they of eventually express their feelings.

The third factor (8.89% of explained variance), named “external support”, included 4 items from the subscales use of instrumental support, self-blame, use of emotional support and humor. These items indicate the quest for external aids to cope with stress in addition to exhibiting a strong emotional component signaled by guilt.

### Retest reliability

The ICC ranged from 0.60 to 0.76 in each of the 3 subscales (Table 3) and the test-retest correlation also ranged from 0.92 to 0.98. Data from these factors in test and retest showed normal distribution with skewness and kurtosis between -0.76 and 0.68. The Cronbach’s alpha value achieved a minimum of 0.60 for factor two and three (Table 3) [35]. The differences between test and retest were not statistically significant with low effect sizes, suggesting good reproducibility of these data as well as low susceptibility to temporal error.

**Table 3:** Reliability, *COPE Breve*, and test-retest comparisons.

Scales	Items		Mean	SD	*Skewness*	*Kurtosis*	α	ICC	p*	Effect size^†^
Religion and positive reframing	22, 24, 7, 27, 12, 20, 25, 17, 14	Test	26.39	5.83	-0.665	0.176	0.77	0.76	0.382	0.06
		Retest	27.16	5.52	-0.611	0.052	0.75	0.75		
Distraction	11, 19, 4, 18, 1, 21,16	Test	8.95	3.06	0.683	-0.146	0.65	0.64	0.365	0.04
		Retest	8.66	3.00	0.403	0.338	0.61	0.61		
External support	10, 26, 5, 28	Test	9.10	3.18	0.184	-0.658	0.63	0.63	0.328	0.06
		Retest	8.73	2.50	0.532	-0.766	0.60	0.60		

*Paired samples t test

^**†**^ Cohen’s d

## Discussion

This study proposes that the Brazilian-Portuguese version of the Brief COPE, i.e., the *COPE Breve*, shows semantic equivalence with the original version in English, after a few changes in wording and format during the cross-cultural adaptation phase. Although the overall *COPE Breve* psychometric properties are fairly suitable, its factorial structure indicates certain inadequacies which necessitate additional discussion and research.

This cross-culturally adapted *COPE Breve* differs somewhat from the Portuguese-Portugal version [11]. This difference was expected because both countries have different cultural contexts, and written and spoken Portuguese in Brazil and Portugal still vary despite reforms to the Portuguese language. Furthermore, our version also contrasts with those versions investigated in other studies with Brazilian university students which adapted the Brief COPE from the Portuguese (Portugal) version [14,15]. In those studies [14,15], students probably had a different socioeconomic background from the participants of this study, who came from the underserved population, because only a minority of people from low-income groups attend university in Brazil. Moreover, one study found that the Brief COPE did not show invariance across Portugal and Brazil; that is, there were differences in the Brief COPE properties when comparing students from those countries [14].

The reliability of the scale concerning the 28 items of *COPE Breve* was satisfactory, but the specific analysis for its 14 subscales showed inconsistencies not detected in the original version. The results of the test-retest correlation indicated that the stability of the *COPE Breve* is satisfactory because most of the scores obtained for the total scale and the individual items exceed 0.75, which is considered excellent [41]. However, the skewness values greater than +1 in the scales substance use, behavioral disengagement and humor show that they have an asymmetric and positively skewed distribution; namely, the right tail of the distribution is longer than the left. This means that few people responded that they use these coping strategies; other study with minority pregnant women also found a few reports on substance use [29]. Then, we can hypothesize that the respondents either really did not routinely use these strategies, or that they gave socially desirable answers; that is, they may not have wanted to admit that they adopt strategies that are not well socially and culturally accepted.

Overall, *COPE Breve* presented acceptable internal consistency, but only four subscales from the original Brief COPE [7] showed a Cronbach’s alpha greater than 0.6: substance use, instrumental support, behavioral disengagement and religion. This suggests that two items of each of the other ten scales did not correlate. Indeed, the 14 subscales are theoretically based and were derived from the "full" COPE instrument, which contained four items per subscales [6]. In Brief COPE, Carver [7] sought to maintain 14 COPE subscales, but now with 2 items per subscale. He also found alpha coefficients under 0.60 in some subscales: acceptance, denial and venting [7]. In this sense, Carver argues that people deal with stressful situations through a list of mutually exclusive coping actions that may sometimes include acceptance, and at other times denial, for example [6].

In this study, the EFA produced a revised 20-item version with a 3-factor solution named “religion and positive reframing”, “distraction” and “external support”. Psychometric properties of the *COPE Breve* with three factors were appropriate. Using EFA, Carver also found that the structure of the Brief COPE has 9 factors [7]. Other studies that also performed EFA on the Brief COPE found 4 to 12 factors [8,11–13,15,18,22,25,26,29,30]. However, there were three investigations that also found a 3-factor structure in the EFA [20,21,23]. Interestingly, one study noted that, as the Brief COPE measures ways of coping in both microscopic (i.e., 14 coping subscale strategies) and macroscopic (i.e., five coping dimensions) ways, its latent structure can be complex and “an aggregate measure represented by the higher-order factors could be an appropriate alternative” [26]. Higher-order factors or coping dimensions are the following: support seeking, problem solving, cognitive restructuring, avoidance and distraction [42].

In the present study, some items from different subscales tended to cluster into factors resembling those of the original Brief COPE [7]. The use of positive reframing, acceptance, religion and planning formed a single factor (factor 1 here), as did self-distraction and substance use (factor 2). However, in our study, items from the subscales active coping, denial, venting, self-blame use of emotional support, behavioral disengagement and use of instrumental support loaded on to different factors or had to be excluded. As Carver also found [7], factors in our study included items from various subscales; he noted that such a dialectic tension between opposing forces within the same factor demands more thorough research. Thus, two of the factors were consistent with three of the five coping dimensions [42]: distraction–factor 2; support seeking–factor 3. Factor 1 comprised the dimensions “cognitive restructuring” and “problem solving”, beside religion.

Nevertheless, the *COPE Breve* presented a good model fit in the EFA but the explained variance was under 50%, thus suggesting that there may be other dispositional coping mechanisms related to Brazilian culture that were not addressed by the adapted instrument. The same finding was observed in a study of people living with HIV/AIDS in South India [13]. Another limitation of the study is the presence of complex items in factors 2 and 3, as well as the low communality values in many items (below 0.5) that might hinder the recovery of population factors. Importantly, the analysis was based on answers provided by individuals recruited at a particular set of institutions; the results may need to be reinforced by other studies which apply the Brief COPE to the general population of Brazil.

Perhaps one of the problems that most profoundly affects the comparison of results between different studies investigating the structure of the Brief COPE is the lack of consistency in its application. The literature reports participants from different socioeconomic status and levels of education [12–15,17–30]. Sometimes, studies either add new questions [21] or omit a few of the previously validated instrument [29]. Moreover, one study used a 5-point instead of the original 4-point Likert scale [14] in an attempt to improve the CFA model fitness.

Several hypotheses should be tested in future studies to account for the aforementioned divergence in results for the factor analysis. One such hypothesis concerns socioeconomic and gender issues. The participants of the present study were mostly adult women from an underprivileged population, whereas those in other studies were students [7,11,14,15,22,26], sick people [13,18,20,23,25,28,30] or others. Thus, one might assume that the present sample was older and more heterogeneous due to its education and income levels, a finding that was not duly investigated here. However, another study with French college students reported on the gender-invariance of the Brief COPE’s hierarchical structure [26]. Indeed, our sample could resemble that from the investigation of 134 Kenyan caregivers, mostly women, for a family member living with HIV/AIDS, but they were asked about situational coping and the authors performed principal component analysis [19].

All in all, because the *COPE Breve* was elaborated following a systematic procedure for cross-cultural adaptation based on a broad encompassing model that takes sociocultural differences into account in the instrument adaptation [32], the variety exhibited by this Brazilian population might similarly characterize diversity in coping compared with other countries. Also, the participants in this study had not undergone any patently stressful situations; rather, they were instructed to imagine one and to report their dispositional coping. Therefore, future studies should include and compare Brazilian individuals in different contexts and situations to further explore the dimensionality of the *COPE Breve* using CFA, for example. Furthermore, the item response theory might provide additional means of validating the internal structure of the *COPE Breve*.

## Conclusions

This culturally adapted version of the Brief COPE (*COPE Breve*) to Brazilian Portuguese does not reveal good psychometric properties for the 14 subscales reported in the original Brief COPE. Instead, *COPE Breve* presents a 3-factor structure in this initial study, for which psychometric properties were appropriate. This 3-factor model could be the basis for another Brazilian study with the *COPE Breve* using CFA.

## Supporting Information

S1 Text The *COPE Breve*. (PDF)
