# Supplementary material for: Controversies Regarding the Psychometric Properties of the Brief COPE: The Case of the Brazilian-Portuguese Version “COPE Breve”
Source: PLoS One. 2016 Mar 23;11(3):e0152233. doi: 10.1371/journal.pone.0152233 (PMC4805194; doi:10.1371/journal.pone.0152233)
Supplement: S1 Text — (PDF) [file pone.0152233.s001.pdf]

COPE Breve*

Estamos interessados em saber a maneira como você está tentando lidar com uma situação difícil. Cada item abaixo refere-se a uma maneira específica de lidar com essa situação. Avalie cada item separadamente, respondendo com as opções 1, 2, 3 ou 4 dentro dos parênteses. Responda as questões da forma mais sincera possível.

1 = Não tenho feito de jeito nenhum
2 = Tenho feito um pouco
3 = Tenho feito mais ou menos
4 = Tenho feito bastante

( ) 1. Tenho me dedicado ao trabalho ou outras atividades para me distrair.
( ) 2. Tenho concentrado meus esforços para fazer alguma coisa em relação à situação na qual me encontro.
( ) 3. Tenho dito a mim mesmo(a): "isto não é real".
( ) 4. Tenho consumido álcool ou outras drogas/medicamentos para me sentir melhor.
( ) 5. Tenho recebido apoio emocional de outras pessoas.
( ) 6. Estou desistindo de enfrentar a situação.
( ) 7. Tenho tomado alguma atitude para tentar melhorar a situação.
( ) 8. Tenho me negado a acreditar que essa situação tenha acontecido.
( ) 9. Tenho dito coisas para extravasar meus sentimentos desagradáveis.
( ) 10. Tenho recebido ajuda e conselhos de outras pessoas.
( ) 11. Tenho consumido álcool ou outras drogas/medicamentos para me ajudar a superar a situação.
( ) 12. Tenho tentado enxergar a situação de outra forma para fazê-la parecer mais positiva.
( ) 13. Tenho me criticado.
( ) 14. Tenho tentado criar uma estratégia em relação ao que fazer.
( ) 15. Tenho recebido conforto e compreensão de alguém.
( ) 16. Estou desistindo de tentar enfrentar a situação.
( ) 17. Tenho tentado enxergar algo de bom no que está acontecendo.
( ) 18. Tenho feito piadas sobre a situação.
( ) 19. Tenho feito coisas para pensar menos na situação como ir ao cinema, ver TV, ler, sonhar acordado(a), dormir ou ir às compras.
( ) 20. Tenho aceitado a realidade do fato acontecido.
( ) 21. Tenho expressado meus sentimentos negativos.
( ) 22. Tento tentado encontrar conforto em minha religião ou crenças espirituais.
( ) 23. Tenho tentado obter conselho ou ajuda com outras pessoas sobre o que fazer.
( ) 24. Tenho aprendido a conviver com esta situação.
( ) 25. Tenho pensado bastante sobre os passos que irei dar.
( ) 26. Tenho me culpado pelas coisas que aconteceram.
( ) 27. Tenho orado ou meditado.
( ) 28. Tenho ridicularizado a situação.

**Versão adaptada para o Português-Brasil por Brasileiro SV. Adaptação transcultural e propriedades psicométricas do Cope Breve em uma amostra brasileira. Goiânia-GO, Brasil, 2012.Dissertação de Mestrado. Programa de Pós-Graduação em Ciências da Saúde da Universidade Federal de Goiás. 84p.*
